# The role of mitophagy in innate immune responses triggered by mitochondrial stress

**DOI:** 10.1186/s12964-020-00659-x

**Published:** 2020-11-25

**Authors:** Yinjuan Song, Yang Zhou, Xiangmei Zhou

**Affiliations:** 1 Key Laboratory of Animal Epidemiology and Zoonosis, Ministry of Agriculture, National Animal Transmissible Spongiform Encephalopathy Laboratory, College of Veterinary Medicine, China Agricultural University, Beijing, 100193 China; 2 College of Animal Science, Southwest University, Chongqing, 402460 China; 3 Immunology Research Center, Medical Research Institute, Southwest University, Chongqing, China

**Keywords:** Mitophagy, Mitochondrial stress, Innate immunity, Infection, Mitophagy mechanisms

## Abstract

**Abstract:**

Mitochondria are important cellular organelles involved in many different functions, from energy generation and fatty acid oxidation to cell death regulation and immune responses. Accumulating evidence indicates that mitochondrial stress acts as a key trigger of innate immune responses. Critically, the dysfunctional mitochondria can be selectively eliminated by mitophagy. The elimination of dysfunctional mitochondria may function as an effective way employed by mitophagy to keep the immune system in check. In addition, mitophagy can be utilized by pathogens for immune evasion. In this review, we summarize how mitochondrial stress triggers innate immune responses and the roles of mitophagy in innate immunity and in infection, as well as the molecular mechanisms of mitophagy.

Video Abstract

**Graphical abstract:**

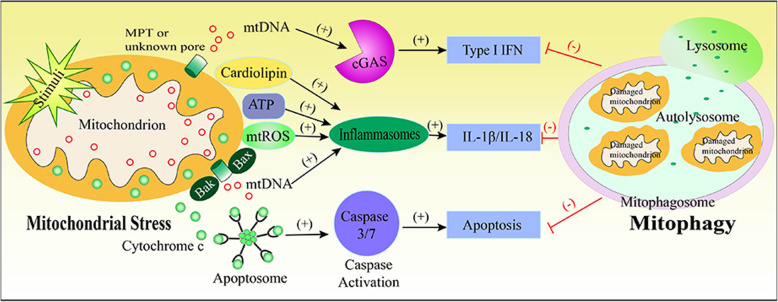

## Introduction

The innate immune system is the first line of defense against pathogen infection. Pathogens are initially sensed by pattern-recognition receptors (PRRs) of the innate immune system, which bind to pathogen-associated molecular patterns (PAMPs), including the structural components, nucleic acids and proteins of pathogenic microorganisms [1]. The PRRs include four families: Toll-like receptors (TLRs), nucleotide oligomerization domain (NOD)-like receptors (NLRs), C-type lectin receptors (CLRs) and retinoic acid-inducible gene I (RIG-I)-like receptors (RLRs) [2, 3]. Ligand binding initiates activation of PRRs, resulting in the activation of nuclear factor-κappa B (NF-κB), mitogen-activated protein kinases (MAPKs), inflammasomes and interferon regulatory factors (IRFs), and subsequent production of pro-inflammatory cytokines, chemokines, type I interferon (IFN) and co-stimulatory molecules [1]. Multiple studies have established crosstalk between innate immune signals and mitochondria.

Mitochondria are dynamic, double-membrane-bound eukaryotic organelles that originated from an ancient α-proteobacterium more than 1.5–2.5 billion years ago [4–6]. Mitochondria maintain their own genetic material, mitochondrial DNA (mtDNA), which is a small, double-stranded circular molecule that only encodes 13 necessary protein subunits of the oxidative phosphorylation system (OXPHOS), and about 99% of mitochondrial proteins are nuclear DNA encoded [7]. Mitochondria perform multiple functions in cells, which are the energy factory of the cell. In addition to energy generation, their crucial roles in metabolism include amino acid and fatty acid metabolism and biosynthesis of heme, hormones and iron-sulfur clusters. Moreover, mitochondria are involved in the regulation of apoptosis and calcium homeostasis, and reactive oxygen species (ROS) signaling [8]. Furthermore, mitochondria have been indicated to have various functions in innate immune pathways. They orchestrate signaling and effector responses to enhance immune cell activation and antimicrobial defense, and elicit inflammation in response to intracellular and extracellular stimuli [9]. Mitochondrial dysfunction involved in various processes closely related to abnormalities of the immune system [10]. Hence, it is important to maintain sufficient healthy mitochondria to keep the immune system in check [11].

Mitophagy is utilized by eukaryotic cells to eliminate damaged mitochondria and preserve healthy mitochondria. It is one of the major pathways of mitochondrial quality control, which monitors the function of mitochondrial and provokes a repair response when damage is discerned [12]. In 1966, mitophagy was first observed in mammalian cells by early electron microscopy studies [13]. Mitophagy is a vital selective autophagy process and subdivided into ubiquitin (Ub)-dependent or Ub-independent (receptor-dependent) pathways [14]. Accumulating evidence implicates that mitophagy keeps the immune system in check by removing stressed mitochondria [11].

Mitochondria are increasingly recognized as pivotal hubs in innate immune responses, and mitochondrial damage-associated molecular patterns (DAMPs) are key triggers in the activation of innate immune responses following a variety of stimuli that include infection, tissue damage and metabolic dysregulation [15]. Moreover, mitophagy is critical for maintaining mitochondrial function and homeostasis [16]. In this review, we summarize the emerging researches on the function of mitophagy in innate immune responses triggered by mitochondrial stress, with a specific focus on the molecular mechanisms of mitophagy and the role of mitophagy in type I IFN production, IL-1β responses and apoptosis.

## Mitochondrial stress acts as a trigger of innate immune responses

### Mitochondrial stress

Mitochondrial stress can be caused by various insults from environment (e.g., radiation, toxic chemicals), genetic mutations (e.g., mutations in genes for metabolic processes or repair pathways), pathogen infection (e.g., virus, bacteria) [17] and spontaneous (e.g., ROS generated as a byproduct of electron transport) [12]. These insults damage mitochondrial proteins, lipids, and DNA, resulting in loss of mitochondrial membrane integrity and membrane potential, metabolic dysfunction, alterations in energy intermediates or impairment of mitochondrial translation [12, 18, 19]. To cope with these challenges, mitochondria continuously crosstalk with the nucleus and the cytosol, activating quality-control proteases [20], lipases [21], mitochondrial unfolded protein response [22], apoptosis [23], mtDNA repair enzymes [24, 25], mitochondrial morphology transitions [26, 27] and mitophagy [14] to restore proper mitochondrial function.

Specifically, mitochondrial stress is characterized by impaired OXPHOS and metabolism, loss of membrane potential and increased levels of mitochondrial ROS (mtROS), etc. These processes effectively disrupt mitochondrial membrane integrity, leading to the release of DAMPs into the cytosol or extracellular environment. Mitochondrial DAMPs include mtDNA, mtROS, N-formylated peptides and cardiolipin. These mitochondrial DAMPs can activate PRRs of the innate immune system, then trigger a wide range of inflammatory responses, including inflammasome activation, production of type I IFN and pro-inflammatory cytokines and chemokines [10]. Moreover, it has been shown that mitochondrial-derived molecules (i.e., cytochrome c) are involved in apoptosis [28]. In this scenario, we will discuss how mitochondrial stress triggers inflammatory responses (i.e., IL-1β, type I IFN responses) and how mitochondria control cell death.

### Mitochondrial stress and inflammasome

Inflammasomes are an essential part of the host innate immune system. They are cytoplasmic multiprotein complex composed of the innate immune sensor molecule and the effector molecule (i.e., pro-caspase-1). Innate immune sensors including Nod-like receptor CARD domain containing 4 (NLRC4), Nod-like receptor pyrin domain containing 3 (NLRP3) or absence in melanoma 2 (AIM2) [29–32]. The requirement of adaptor molecules (i.e., apoptosis-like speck protein, or ASC) relies on the presence or absence of matching domains within the sensor and effector molecules. Some inflammasomes may require adaptor molecules that bridge the interactions of protein-protein to ultimately elicit inflammasome assembly [29, 32, 33]. The sensor, adaptor, and effector molecules of inflammasome form a large cytosolic multiprotein complex upon stimulated by its activators (e.g., pathogen infection or sterile injury), leading to the self-cleavage and activation of pro-caspase-1. Then, active caspase-1 proteolytically processes pro-interleukin-1β (pro-IL-1β) and pro-IL-18, into their biologically active forms (namely IL-1β and IL-18), respectively [29, 30, 32, 33].

The NLRP3 inflammasome is the most extensively studied inflammasome and is activated by a wide spectrum of stimuli including microbial infection, bacterial pore-forming toxins, aluminum hydroxide, silica crystals, monosodium urate, extracellular ATP and asbestos [34–38]. It has been proposed that the activation of NLRP3 is controlled by mitochondria [39]. In 2011, Nakahira et al. found that cytosolic mtDNA contributes to the secretion of IL-1β and IL-18 in response to lipopolysaccharide (LPS) and ATP, and the release of mtDNA into the cytosol depends on the mtROS production [40]. mtDNA is required for NLRP3 inflammasome activation in murine macrophages for depletion of mtDNA (low-dose chronic treatment of ethidium bromide) attenuates IL-1β release and caspase-1 activation [40]. This study first linked mtDNA to the activation of NLRP3 inflammasome. In 2012, NLRP3 was shown to bind to cytosolic oxidatively damaged or oxidized mtDNA (Ox-mtDNA) released during apoptosis causing its activation [41]. This study extended our understanding of the interplay between mtDNA and the NLRP3 inflammasome. The above results were further verified by another approach by which the genetic ablation of mitochondrial transcription factor A (TFAM) in mouse myeloid cells causes more than 95% mtDNA deletion and prevents NLRP3 inflammasome activation [42]. Transfection of Ox-mtDNA in TFAM-ablated macrophages rescued the activity of defective NLRP3 inflammasome, suggesting that Ox-mtDNA is sufficient and necessary for NLRP3 inflammasome activation [42]. In addition to NLRP3, mtDNA may influence other inflammasomes activation: mtDNA bound specifically to NLRC4 immunoprecipitate and transfection of mitochondrial DNA directly activated the NLRC4 inflammasome [43]. Moreover, the mtDNA from patients with type 2 diabetes triggers AIM2 inflammasome-dependent caspase-1 activation and IL-1β and IL-18 secretion in macrophages [44].

In 2010, the generation of ROS proved to be one of the essential elements for NLRP3 inflammasome activation [45]. In 2011, Zhou et al. found that mtROS was essential for NLRP3 inflammasome activation [39]. Although mtROS is essential, it is not sufficient to activate NLRP3. The addition of H_2_O_2_ (the dominant form of mtROS) cannot rescue defective NLRP3 inflammasome activity in TFAM-deleted macrophages [42]. This is in line with the fact that not all mtROS inducers (e.g., TNF) activate NLRP3 inflammasome [29, 30]. Additionally, NLRP3 inflammasome activation is partially triggered by ATP produced by mitochondria released from damaged cells [46]. The depletion of cardiolipin by knockdown of cardiolipin synthase attenuates NLRP3 inflammasome activation by reverse transcriptase inhibitor abacavir, suggesting mitochondrial cardiolipin also triggers NLRP3 inflammasome activation [47, 48]. Furthermore, some mitochondrial proteins also play a crucial role in inflammasome activation. In 2011, the research showed that voltage-dependent anion channel (VDAC) is important for NLRP3 inflammasome activation. Both ROS generation and inflammasome activation are suppressed when mitochondrial activity is dysregulated by inhibition of the VDAC [39]. The VDAC is a component of the mitochondrial permeability transition pore (MPTP) through which large molecules (e.g., mtDNA) are released into the cytoplasm [49, 50]. In 2013, the study suggested that mitochondrial antiviral signaling proteins (MAVS) is required for optimal NLRP3 inflammasome activity. MAVS mediates the recruitment of NLRP3 to mitochondria, promoting the production of IL-1β and the pathophysiologic activity of the NLRP3 inflammasome in vivo [51].

In summary, mitochondria play a key role in the activation of NLRP3 inflammasome. When cells are stimulated by various stress signals, mitochondria release mitochondrial DAMPs (such as mtROS, mtDNA, ATP) into the cytoplasm, which trigger the assembly of cytosolic NLRP3, ASC and pro-caspase-1 [36]. The entry of mitochondrial DAMPs into the cytoplasm may depend on VDAC [49, 50]. The assembled NLRP3 inflammasome is recruited to the outer mitochondrial membrane and interacts with MAVS to promote inflammasome activation and IL-1β production [51]. Although how NLRP3 inflammasome activation is triggered is extensively investigated, the exact mechanism of its assembly and activation still needs further exploration.

### Mitochondrial stress and type I IFN responses

In addition to inducing pro-inflammatory cytokines production, mtDNA accesses into the cytosol or extracellular space during mitochondrial dysfunction, and triggers expression of type I IFN and interferon-stimulated genes (ISGs) in mice and humans [52]. Although some of the mechanistic details are still unclear, the identification of mtDNA as a DAMP that induces type I IFN production is crucial for understanding the pathology of infectious and non-infectious diseases involving mitochondrial dysfunction [53]. In this section, we will discuss the roles of mtDNA in type I IFN responses.

Cytosolic mtDNA engages cyclic guanosine monophosphate–adenosine monophosphate synthase (cGAS) activation and subsequently elicits type I IFN production. cGAS is a cytosolic nucleotide synthase that binds DNA, and catalyzes ATP and GTP to synthesize cyclic guanosine monophosphate–adenosine monophosphate (cGAMP). Then, cGAMP acts as a second messenger to bind and activate the stimulator of interferon genes (STING) [54, 55]. STING further activates TANK-binding kinase 1 (TBK1) via its C terminal PLPLRT/SD motif [56]. TBK1 promotes IRF3 dimerization and translocation into the nucleus, where it triggers the expression of type I IFN and various ISGs [57, 58]. Specifically, it has been demonstrated that cGAS functions as the main sensor of viral and bacterial DNA in the cytoplasm of infected cells [59–61], such as *Mycobacterium tuberculosis* (Mtb) infected bone marrow-derived macrophages (BMDM) cGAS senses Mtb DNA in the cytosol and triggers the expression of type I IFN. Recent evidence indicates that mtDNA may also serve as an endogenous cGAS ligand in certain conditions. For instance, mitochondrial stress provoked by Mtb infection results in mtDNA leaking into the cytoplasm and cytosolic mtDNA contributes to IFN-β production by activating the cGAS-STING pathway [17]. Moreover, West and colleagues found that mouse embryonic fibroblasts (MEFs) from the TFAM heterozygous knockdown (TFAM^+/−^) mice, exhibit disruption of mtDNA stability, which leads to mtDNA release into the cytosol, where it engages the DNA sensor cGAS and promotes STING-IRF3-dependent signaling to elevate ISGs expression and potentiate type I IFN responses [62].

Furthermore, previous studies demonstrated that mtDNA release during Bcl-2 associated-X protein (Bax) and BCL-2 homologous antagonist/killer (Bak)-mediated apoptosis elicits cGAS-STING signaling and induces type I IFN production. Inhibition of apoptotic caspase-9/− 3/− 7 cascade enhances the type I IFN responses triggered by cytosolic mtDNA, suggesting that apoptotic caspase-9/− 3/− 7 cascade suppress mtDNA-mediated type I IFN production during the apoptosis process [63, 64]. Recently, apoptotic caspase-3 was shown to inhibit type I IFN production via cleaving and inactivating cGAS, MAVS and IRF3 [65]. Notably, active caspase-1 was also shown to cleave cGAS in macrophages and inhibit type I IFN expression [66]. These results collectively indicate that the activation of cGAS triggered by host-derived mtDNA is likely to be a strictly regulated process to hinder uncontrolled type I IFN production that might otherwise cause pathological disease (i.e., autoimmunity disease).

Toll-like receptor 9 (TLR9) was the first identified nucleic acid-sensing TLR and recognizes hypo-methylated cytosine-guanosine (CpG) motifs in the DNA. It is expressed primarily in monocytes, macrophages, plasmacytoid dendritic cells (pDCs) and B lymphocytes [67]. In addition to recognizing bacterial or viral DNA, mounting literature supports the notion that mtDNA is a potent ligand for TLR9, for bacterial DNA and mtDNA share hypo-methylated CpG motifs that can be recognized by TLR. For example, the plasma from mice and patients with nonalcoholic steatohepatitis (NASH) contains high levels of mtDNA that has the ability to activate TLR9 [68]. And, mtDNA contributes to the initiation of sterile systemic inflammatory response syndrome (SIRS) via activating the TLR9/NF-kB pathway and inducing pro-inflammatory cytokines expression [69]. In addition to eliciting pro-inflammatory responses, extracellular oxidized mtDNA has been shown to induce the TLR9- and IRF7-dependent production of IFN-α to augment type I IFN responses in pDCs [70].

From the mentioned above, mtDNA appears to be a potent ligand of the cGAS and TLR9. mtDNA influences type I IFN responses and pro-inflammatory responses in multiple pathological states, including infectious diseases, autoimmunity, and other illnesses in which mitochondrial integrity is damaged. Even though previous studies proposed mitochondria-derived vesicles (MDVs) [52], MPTP opening [40, 71] and Bax/Bak proteins oligomerization [72] have been associated with the release of mtDNA into the cytoplasm, the precise mechanisms underlying mtDNA escape from damaged mitochondria remain unclear.

### Mitochondria control cell death

Mitochondria play a crucial role in the regulation of apoptotic cell death. These organelles not only modulates intrinsic apoptosis triggered by alterations of intracellular homeostasis but also participates in the regulation of the extrinsic apoptotic response to external stimuli [73]. Besides apoptosis, mitochondria also control non-apoptotic types of programmed cell death, including regulated necrosis [74]. The mechanisms of mitochondria control cell death are extensively reviewed elsewhere [4, 28]. The main factors carrying out apoptosis include Bax and Bak, which trigger the outer mitochondrial membrane (OMM) permeabilization upon activation, leading to the irreversible release of intermembrane space proteins (e.g., cytochrome c), subsequent activation of caspase-9/− 3/− 7 and apoptosis [75]. Apoptosis is an important innate cellular defense mechanism that prevents the growth of intracellular microbes, including virulent Mtb [76]. The crosstalk between mitochondrial apoptosis pathway and various pathogens infection was reviewed in 2020 by Varnesh Tiku et al. [77]. Here, we focus on the mechanisms of mycobacterium-induced apoptosis and necrosis, and the role of mitochondria in it.

Pathogenic intracellular bacteria have evolved multiple strategies to target host cells to either inhibit apoptosis to maintain their survival and growth or promote apoptosis. The previous study suggested that Mtb differentially regulates myeloid cell leukemia-1(Mcl-1) and Bax expression through peroxisome proliferator-activated receptor (PPAR) gamma to limit apoptosis [76]. Mtb heparin-binding hemagglutinin (HBHA) targets to mitochondria and then caused mitochondrial damage and oxidative stress, which eventually lead to Mtb infected or HBHA-treated macrophages apoptosis [78]. In addition to Mtb, *Mycobacterium avium* MAV2054 protein induces caspase-dependent macrophage apoptosis by targeting mitochondria. The apoptotic response and ROS production were significantly increased and mitochondrial transmembrane potential (ΔΨm) was decreased in macrophages infected with *Mycobacterium smegmatis* expressing MAV2054 [79]. A recent study reported that *M. bovis* infection upregulates the expression of the Kallikrein 12 (KLK12), and knockdown of KLK12 results in a significant downregulation of autophagy and apoptosis in *M. bovis*-infected BMDMs. Furthermore, KLK12-mediated regulation of apoptosis involves BAX/Bcl-2/cytochrome c/caspase 3 pathways [80]. *Mycobacterium fortuitum* infection induces Ca^2+^ mobilization from the endoplasmic reticulum (ER) to mitochondria, leading to increased mitochondrial Ca^2+^ load, MPTP opening, altered mitochondrial membrane potential (ΔΨm), cytochrome c release, and eventually activation of the caspase-9/− 3 mediated apoptosis in fish macrophage [81].

Mycobacterial infection leads to apoptosis or necrosis depends on differences in mitochondrial membrane perturbation induced by attenuated or virulent strains. The virulent H37Rv promotes necrosis via inducing substantial alterations to mitochondrial transmembrane potential (ΔΨm), while the attenuated H37Ra infection predominantly leads to apoptosis [82]. A previous study reported that Mtb Rv2626c gene is associated with the host cell necrosis. When Rv2626c gene is deleted from the genome of Mtb, THP-1 cells undergo less necrosis following infection with Rv2626c gene-deleted Mtb, and overexpression of Rv2626c promotes the host cell necrosis at early time points [83]. However, the mechanism by which mycobacterium promotes necrosis has not been fully elucidated. In summary, mitochondria have an important role in intrinsic apoptosis as well as necrosis.

**Fig. 1:**
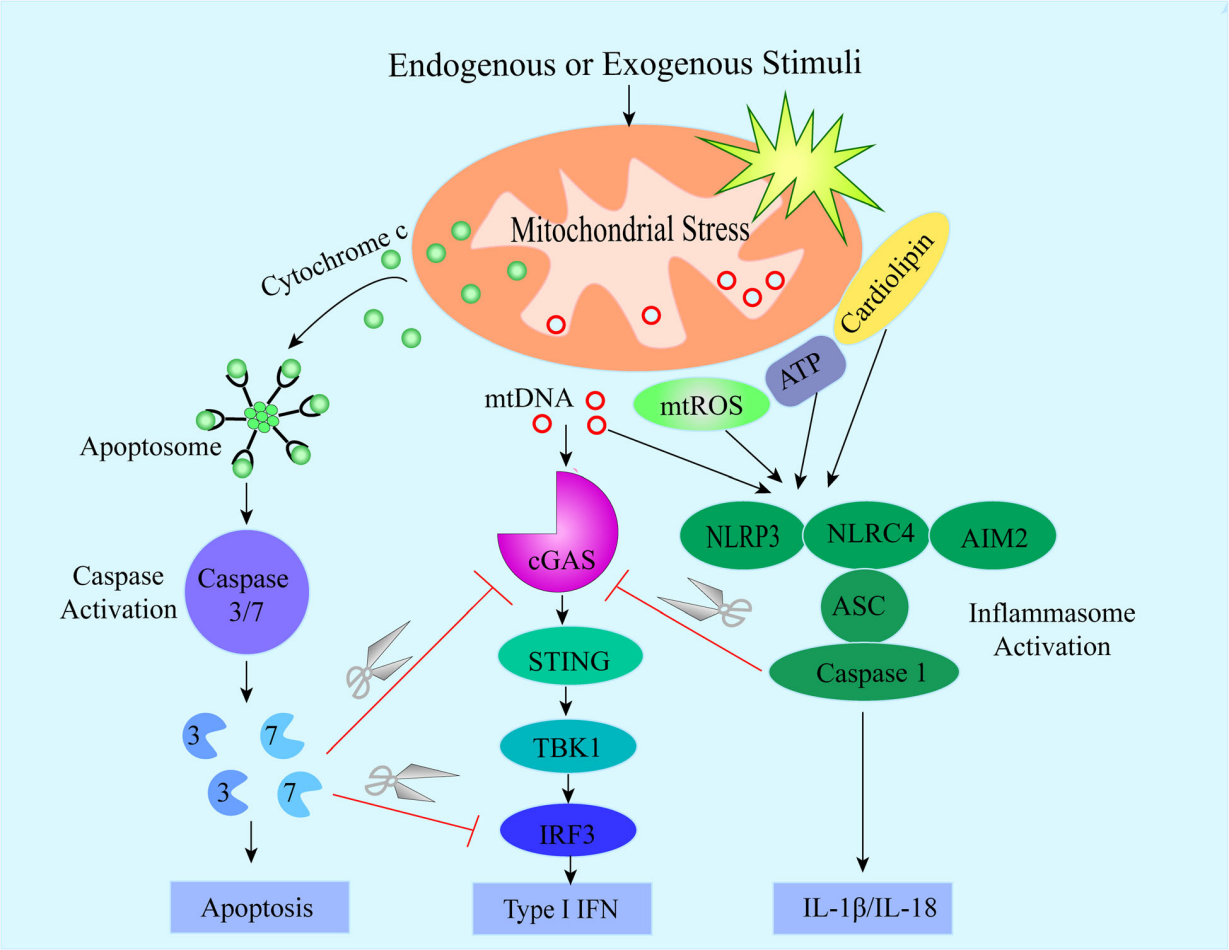
Mitochondrial stress acts as a trigger of innate immune responses. Cell and tissue stress initiated by endogenous or exogenous stimuli can directly or indirectly cause mitochondrial stress. Mitochondrial stress leads to mtROS, mtDNA and ATP release into the cytosol or extracellular milieu, cardiolipin externalization and cytochrome c release into the cytosol. Cytosolic mtDNA activates the cGAS-STING pathway, resulting in the production of type I IFN. Moreover, mtROS, mtDNA, cardiolipin and ATP engage in the activation of inflammasome leading to IL-1β and IL-18 processing and secretion. Cytosolic cytochrome c triggers activation of apoptotic caspase-3/− 7, and which inhibits type I IFN production via cleaving cGAS and IRF3. Similarly, caspase-1 also can cleave cGAS to prevent type I IFN expression

As described above, mitochondria play key roles in the innate immune responses and mitochondrial stress causes inflammasome activation, type I IFN production and induction of apoptosis (Fig. 1). Meanwhile, mounting studies demonstrated that mitophagy can prevent inflammasome activation by clearing damaged mitochondria [84–87]. Next, we will summarize the studies on the molecular mechanisms of mitophagy, and the function of mitophagy in innate immune responses triggered by mitochondrial stress.

## Molecular pathways of mitophagy

Autophagy is a process that delivery of cytoplasmic cargo to the lysosome for degradation and highly conserved in eukaryotes. There are at least three different autophagy forms (i.e., chaperone-mediated autophagy, microautophagy, and macroautophagy) which differ in the way of cargo delivered to the lysosome. Macroautophagy (herein referred to as autophagy) is the crucial degradative mechanism adopted by eukaryotic cells to sustain nutrient homeostasis and organellar quality control [88–90]. Autophagy was previously described as a non-selective process in response to starvation. However, it is now clear that autophagy can selectively degrade damaged or dysfunctional organelles, remove aggregated proteins and eliminate intracellular pathogens [91]. Several types of selective autophagy have been described, such as aggrephagy (removal of aggregated proteins), mitophagy (turnover of damaged mitochondria), pexophagy (degradation of peroxisomes), ribophagy (turnover of damaged ribosomes), reticulophagy (degradation of the endoplasmic reticulum) and xenophagy (elimination of pathogens) [90, 91].

The process of autophagy that selectively degrades mitochondria is called mitophagy, which is critical for mitochondrial quality control and the maintenance of normal cellular physiology. Cells possess multiple mitophagy mechanisms, and different stimuli promote mitophagy through multiple signal cascades in different cellular environments [92]. In general, mitophagy regulatory pathways are classified as ubiquitin (Ub)-dependent or Ub-independent (receptor-dependent) [14].

### Pink1–Parkin-mediated mitophagy

The phosphatase and tensin homologue (PTEN)-induced putative kinase 1 (Pink1)– parkin RBR E3 ubiquitin protein ligase (Parkin) mediated mitophagy is the most extensively studied. Ub-dependent pathway is employed to clear damaged mitochondria and is generally related to the alteration of mitochondrial transmembrane potential [93]. Pink1 is a mitochondrial Ser/Thr kinase, encoded in nuclear DNA, exists as a precursor form in the cytosol, and then transported into mitochondria [94]. Under physiological conditions, Pink1 is transported into the inner mitochondrial membrane (IMM), and cleaved by several IMM-resident proteases include presenilin-associated rhomboid-like (PARL). Cleaved Pink1 is retro-translocated into the cytoplasm and subsequently degraded by the proteasome through the N-end rule pathway [94]. Following mitochondrial membrane potential dissipation, the translocation of cytosolic Pink1 into the mitochondrial matrix is prevented, leading to the stabilization of Pink1 on the outer membrane of the damaged mitochondria [94]. Concomitantly, Pink1 recruits Parkin to the mitochondrial surface and triggers its E3 ligase activity through a circuit of modifications including phosphorylation of both Parkin and ubiquitin [95]. Subsequently, Parkin mediates poly-ubiquitination of several OMM proteins including VDAC1 and mitofusin 1/2 (MFN1/2). Polyubiquitinated proteins are identified by several adaptor molecules, including SQSTM1/p62, optineurin (OPTN), and nuclear domain 10 protein 52 (NDP52, also known as CALCOCO2), facilitating their recognition by light chain 3 (LC3) and mitophagosome (mitochondria-specific autophagosome) formation [96]. Importantly, phosphorylated ubiquitin (pSer65-Ub and pSer57-Ub), functions as potent Parkin activator, leading to enhanced ubiquitination of proteins at the OMM and initiation of mitophagy [97–99]. Therefore, Pink1-mediated phosphorylation and Parkin-mediated ubiquitination form a feedforward mechanism of mitophagy [100, 101].

Notably, the negative regulatory mechanisms against Parkin-mediated ubiquitination have been reported, with the discovery of several deubiquitinases including USP8, USP15, USP30 and USP35 that are able to cause the deubiquitination of Parkin and/or OMM proteins [102–107]. Furthermore, PTEN-Long (PTEN-L) is a novel negative regulator of mitophagy through its protein phosphatase activity inhibiting phosphorylated ubiquitin (pSer65-Ub). Meanwhile, it can effectively prevent Parkin mitochondrial translocation, reduce Parkin phosphorylation, maintain its inactive conformation, and inhibit its E3 ligase activity [108]. Therefore, the delicate balance between ubiquitination and deubiquitination events regulates cellular homeostasis, suggesting poly-Ub chains may serve as “eat me” signals to damaged mitochondria.

Pink1–Parkin-mediated mitophagy pathway interplays with other mitochondrial quality control mechanisms, such as mitochondrial dynamics, to maintain cellular homeostasis. Mitochondrial dynamics refers to the dynamic changes of mitochondria undergoing fission and fusion to maintain their shape, distribution and size. Mitochondrial fusion is assured by Mfn1 and Mfn2 and optic atrophy 1 (OPA1), which mediate OMM and IMM fusion, respectively. Mitochondrial fission is regulated by the cytosolic GTPase-protein dynamin-related protein 1 (DRP1) [109]. Pink1 serves as a pro-fission signal, which indirectly activates DRP1 in response to damage, and promotes fission of dysfunctional mitochondria, thereby segregating damaged mitochondria for mitophagy [110]. The ubiquitination of Mfn1 and Mfn2 are induced by Parkin during membrane depolarization and cause their degradation in a proteasome- and p97 - dependent manner [111]. In turn, Mfn2 mediates Parkin recruitment to damaged mitochondria in a Pink1-dependent manner. Depletion of Mfn2 in mouse cardiac myocytes inhibits depolarization-elicited translocation of Parkin to mitochondria and prevents mitophagy [112]. Recent studies suggest that Mfn2 is a mitochondria-ER tether that acts as a suppressor of mitophagy through the ability to link OMM to ER, which may limit the approachability of other ubiquitinated substrates to Pink1 and Parkin [113, 114].

In addition to Parkin, other E3 ubiquitin ligases are also implicated in mitophagy regulation, including HUWE1 [115], autocrine motility factor receptor (AMFR/ Gp78) [116], SMAD-specific E3 ubiquitin-protein ligase 1 (SMURF1) [117], mitochondrial E3 ubiquitin-protein ligase 1 (MUL1) [118], seven in absentia homolog 1 (SIAH1) [119], and Ariadne RBR E3 Ub protein ligase 1 (ARIH1), also known as HHARI [120] and MARCH 5 [121]. Once recruited on the OMM, they generate ubiquitin chains, triggering recruitment of several autophagy adaptor proteins, including OPTN, NDP52 and p62. Autophagy adaptor proteins interact directly with LC3 through their LC3-interacting region (LIR) motifs, initiating mitophagosome formation.

### Ub-independent mitophagy

In addition to Ub-dependent mitophagy, several LC3-interacting region (LIR) containing mitophagy receptors can directly induce Ub-independent mitophagy, including BCL2 interacting protein 3 (BNIP3) [122], BCL2 interacting protein 3 like (BNIP3L/NIX) [123], FUN14 domain-containing protein 1 (FUNDC1) [124], BCL2 like 13 (BCL2L13) [125], and FK506 binding protein 8 (FKBP8) [126] in mammals. These mitophagy receptor proteins are located on the OMM and interact directly with LC3 via their LIR motifs, mediating mitochondrial clearance. Of note, the recent research identified that NLRX1 is a novel mitophagy receptor for *Listeria monocytogenes* (*L. monocytogenes*)–induced mitophagy [127]. NLRX1 is the only mitochondrially localized member of the NLR family [128, 129], which contains an LIR motif for LC3 binding that was essential for *L. monocytogenes*–induced mitophagy.

BNIP3, NIX and BCL2L13 belong to the BCL2 family with pro-apoptotic activity. In addition to its apoptotic functions, BNIP3, NIX and BCL2L13 are also implicated in the initiation of autophagy. In erythroid cells, NIX is required for a specialized type of autophagy that targets mitochondria for elimination. Similarly, BNIP3 regulates mitophagy in response to hypoxia [130]. Bcl2L13 has been shown to induce mitochondrial fission and mitophagy in mammalian cells [125]. BNIP3- and NIX-mediated mitophagy promotes the generation of natural killer cell memory [131]. Homodimerization of BNIP3 is a requirement for its interaction with LC3 and this interaction is regulated by the phosphorylation on Ser17 and Ser24 near the LIR motif [132, 133]. NIX-mediated mitophagy leads to a shift in metabolism towards the glycolysis necessary for retinal ganglion cell (RGC) neurogenesis. Moreover, NIX-mediated mitophagy also regulates the polarization of M1 macrophages, which undergo glycolysis-dependent differentiation during the inflammatory response [134]. NIX-mediated mitophagy protects against ischemic brain injury independent of PARK2, and phosphorylation of NIX at serine 81 is critical for NIX-mediated mitophagy [135]. Furthermore, NIX-mediated mitophagy plays a critical role in CD8^+^ T cell effector memory formation by regulating mitochondrial superoxide dependent hypoxia-inducible factor 1-alpha (HIF1a) protein accumulation and fatty acid metabolism [136]. Recent research reported that NIX and FUNDC1-mediated mitophagy play a critical role in the formation of a functional mature mitochondrial network upon the differentiation of progenitor cells [137].

FUNDC1 is a conserved mitophagy receptor that promotes damaged or superfluous mitochondrial clearance during hypoxia in mammalian cells [124]. FUNDC1 promotes unc-51 like autophagy-activating kinase 1 (ULK1) recruitment to damaged mitochondria. In turn, ULK1 interacts with FUNDC1, phosphorylating it at serine 17, which enhances FUNDC1 binding to LC3 [138]. In addition, FUNDC1 interacts with both DRP1 and OPA1 to coordinate mitochondrial fission or fusion and mitophagy [139]. Further, it has been demonstrated that FUNDC1 is a novel MAM protein required for hypoxia-induced mitochondrial fission and mitophagy [140]. As mentioned above, NIX, BNIP3, and FUNDC1 regulate mitophagy in response to hypoxia. Although the crosstalk between FUNDC1, BNIP3, and NIX has not been fully clarified, their coordinating effects ensure the efficiency of mitochondrial quality control and cellular homeostasis. In addition, FKBP8 is also required for mitophagy under hypoxic stress [141].

Of note, certain IMM proteins have been shown to act as mitophagy receptors under stress conditions, such as cardiolipin and prohibitin2 (PHB2), which interact with lipidated LC3 via LIR motifs to promote the engulfment of defective mitochondria [142, 143]. PHB2 is an IMM protein that plays a role in development, lifespan regulation, and diverse cellular processes, including mitochondrial dynamics [144]. The interaction between PHB2 and LC3 requires OMM rupture and PHB2 cytoplasmic exposure [145]. Cardiolipin is an inner mitochondrial membrane phospholipid. Mitochondrial damage caused the externalization of cardiolipin to the mitochondrial surface. The redistribution of cardiolipin serves as an “eat-me” signal for the elimination of damaged mitochondria from neuronal cells [142].

It is worth mentioning that there is a crosstalk between Pink1–Parkin-mediated mitophagy and Ub-independent mitophagy [146]. It has been identified that PHB2 is required for Parkin-induced mitophagy in mammalian cells and for the clearance of paternal mitochondria after embryonic fertilization in *Caenorhabditis elegans* (*C. elegans*) [143]. The latest research reveals that PHB2-mediated mitophagy depends on the mitochondrial inner membrane protease PARL, which interacts with PHB2 and is activated after PHB2 depletion. Upon mitochondrial membrane depolarization or misfolded protein aggregation, PHB2 depletion causes the cleavage of Pink1 by activating PRAL, which prevents the mitochondrial recruitment of Parkin, ubiquitin and OPTN, leading to an inhibition of Pink1-Parkin-dependent mitophagy [147].

**Fig. 2:**
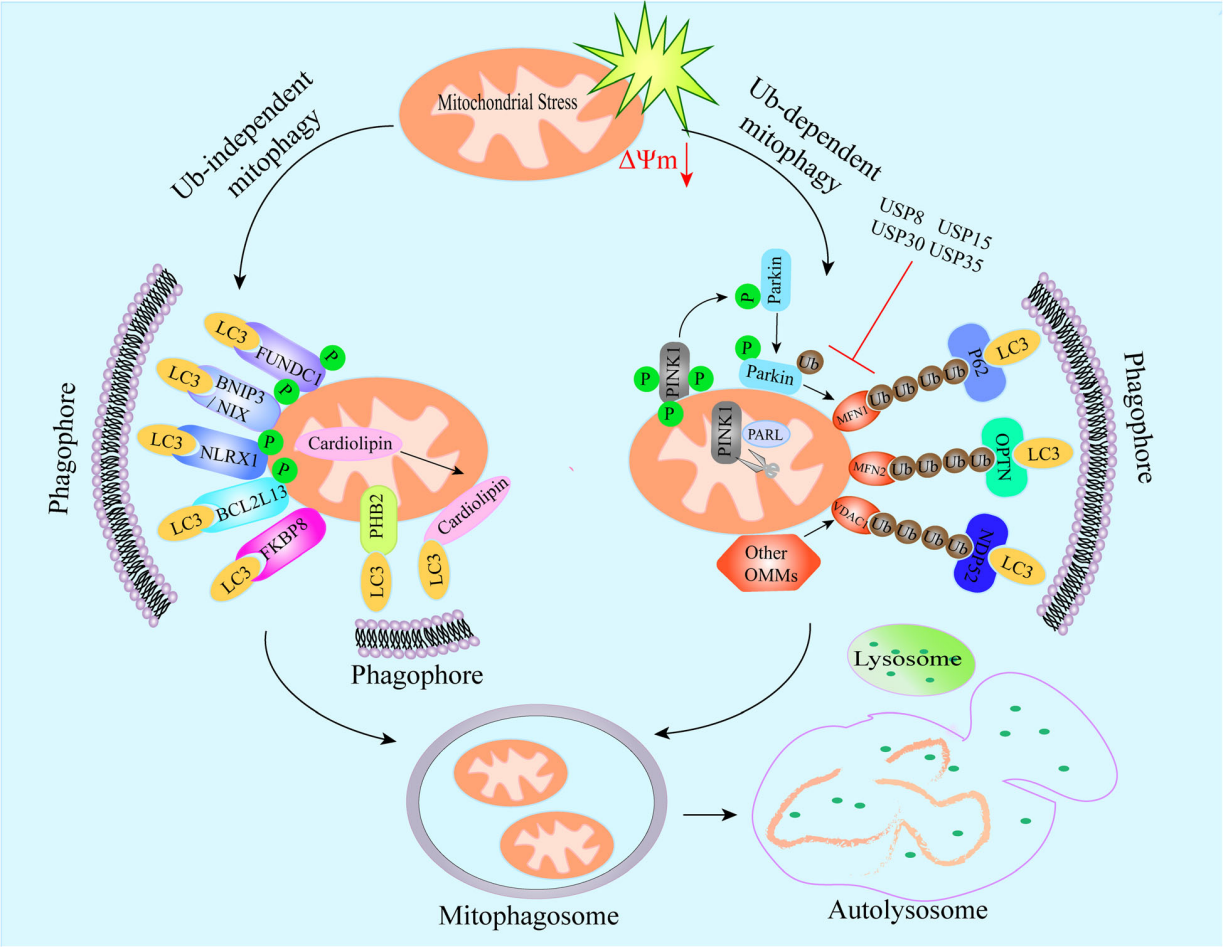
Molecular mechanisms of mitophagy. Ub-dependent mitophagy usually refers to Pink1-Parkin-mediated mitophagy. Upon mitochondrial depolarization, full-length Pink1 accumulates on the OMM and recruits Parkin to the mitochondrial surface. Pink1 triggers Parkin E3 ligase activity through a circuit of modifications including phosphorylation of both Parkin and ubiquitin. Then, parkin ubiquitinates various OMM proteins including VDAC1 and MFN1/2. Polyubiquitinated proteins can be recognized by several adaptor molecules, including p62, OPTN and NDP52. These proteins interact with lipidated LC3 via the LIR motif to promote the encapsulation of damaged mitochondria by the autophagosome. In addition, deubiquitinase such as USP8, USP15, USP30, USP35 can cause the deubiquitination of Parkin and OMM proteins. Ub-independent mitophagy depends on several LIR containing mitophagy receptors, including FUNDC1, BNIP3, NIX, BCL2L13, FKBP8 and NLRX1. Besides, IMM proteins (such as cardiolipin and PHB2) interact with lipidated LC3 through the LIR motif, thereby promoting the phagocytosis of defective mitochondria. Overall, mitophagy adaptors and receptors bind to LC3 bound on the phagophores for further expansion and closure to form mitophagosomes, subsequently, mitophagosomes fuse with lysosomes form autolysosome for mitochondrial degradation

In conclusion, the diversity of mitophagy receptors and adaptor molecules reflects the existence of a compensatory mechanism that can regulate mitochondrial homeostasis in response to a variety of intracellular and extracellular stimulus signals (Fig. 2). The complex interactions between mitophagy mechanisms ensure energy metabolism and cellular homeostasis. Therefore, maintaining mitochondrial function via fine-tuning mitophagy and mitochondrial dynamics is essential for cellular and organismal survival.

## The role of mitophagy in innate immunity and infection

### Mitophagy and inflammasome

As mentioned above, it has been demonstrated that damaged mitochondria play a crucial role in the activation of NLRP3 inflammasome by releasing mitochondrial DAMPs, such as mtROS and mtDNA. Therefore, mitophagy may be a key regulator of NLRP3 inflammasome activation, as it is a process that eliminates damaged or aberrant mitochondria [148]. This notion was experimentally demonstrated in a study showing that the deletion of autophagy protein Beclin 1 and LC3B in BMDM results in the accumulation of defective mitochondria, the increase of mtROS and an increase in mtDNA cytosolic release, which subsequently enhances NLRP3 inflammasome activation [40]. Consistent with these in vitro results, autophagy-deficient mice (Map1lc3b^−/−^ mice) were more sensitive to bacterial sepsis-induced death, which is due to higher IL-1β secretion than wild-type mice [40]. These findings are consistent with an earlier report suggesting that the loss of the autophagy protein autophagy-related 16-like 1 (Atg16L1) elevates endotoxin-induced IL-1β production [149]. The above studies indicate that autophagy inhibits inflammasome activation and subsequent IL-1β secretion. In recent years, studies have further proved that mitophagy can inhibit inflammasome activation. NF-κB restricts NLRP3 inflammasome activation through p62-dependent mitophagy. P62-depleted macrophages display significant mitochondrial damage and excessive IL-1β-dependent inflammation [87]. Moreover, macrophage stress-inducing protein SESN2 inhibits the NLRP3 inflammasome activation through inducing mitophagy [85]. Recently researches showed that OPTN inhibits the activation of NLRP3 inflammasome by enhancing mitophagy [150], and FUNDC1-mediated mitophagy suppresses hepatocarcinogenesis via inhibition of inflammasome activation [151]. Above studies show that mitophagy inhibits NLRP3 inflammasome activation by eliminating damaged mitochondria. Recent study reported that rabbit haemorrhagic disease virus (RHDV) infection induces Pink1-Parkin-mediated mitophagy and NLRP3 inflammasome activation [152]. Moreover, in 2019, Li and colleagues demonstrated that Parkin regulates activation of NLRP3 inflammasome, they found that Parkin deficiency enhances mtROS-mediated NLRP3 inflammasome activation and promotes viral clearance [153]. However, it has been shown that active caspase-1 can inhibit mitophagy by cleaving Parkin [154], suggesting that there may exist a mutual regulation mechanism between inflammasome and mitophagy.

### Mitophagy and type I IFN

Type I IFN, including IFN-α and IFN-β, can activate intracellular antimicrobial programs and influence the development of innate and adaptive immune responses [155]. In recent years, some studies have reported that some viruses can directly or indirectly trigger mitophagy, and control mitophagy process through different strategies, thereby weakening the innate immune response by inhibiting the production of type I IFN or apoptosis, and enabling the virus to promote continuous infection [156]. Such as Measles virus of the Edmonston strain (MV-Edm) triggers p62-mediated mitophagy to suppress the production of type I IFN and enhances viral replication [157]. Parkin is able to recruit the linear ubiquitin assembly complex to mitochondria and abrogates IFN-β production during HBV infection, which indicates that HBV usurps Parkin to impair the cellular antiviral response [158]. Moreover, matrix protein M of human parainfluenza virus type 3 (HPIV3) directly interacts with Tu translation elongation factor mitochondrial (TUFM) and autophagy protein LC3 to induce Pink1-Parkin-independent mitophagy, thereby inhibiting host type I IFN responses [159]. Furthermore, it was demonstrated that glycoprotein (Gn) of the Hantavirus (HTNV) translocates to mitochondria and interacts with TUFM, recruiting LC3B and promoting mitophagy, subsequently inhibits type I IFN responses [160]. The above research indicates that mitophagy contributes to the regulation of type I IFN through clearing dysfunctional mitochondria. In addition, a previous study has unraveled that in the absence of N-terminal truncated isoforms of MAVS, blocking Nix-mediated mitophagy causes the stabilization of full-length MAVS and induces the subsequent secretion of type I IFN and other proinflammatory cytokines [161]. In 2018, a study reported that Parkin and Pink1 mitigate STING-induced inflammation, while STING is a central regulator of type I IFN responses to cytosolic DNA. The study supports a role for Pink1-and parkin-mediated mitophagy in suppressing innate immunity [162]. Hence, mitophagy can regulate type I IFN production by eliminating damaged mitochondria.

### Mitophagy and apoptosis

Mitochondrial dynamics is integrally linked to apoptosis [23]. In 2013, Kim and colleagues reported that hepatitis B virus (HBV) attenuates mitochondrial apoptosis by shifting the balance of mitochondrial dynamics toward fission and mitophagy. They found that silence of Parkin expression induces extensive cytochrome c release from mitochondria to cytoplasm and promotes cleavage of caspase-3 and poly (ADP-Ribose) polymerase (PARP), activation of caspase-3/-7, and promotes intrinsic apoptosis [163]. Similarly, in 2014, it has been shown that HCV promotes Pink1-Parkin-mediated mitophagy, and inhibit host cell apoptosis via eliminating damaged mitochondria to promote viral replication. Depletion of Drp1 and Parkin caused massive cytochrome c release into cytosol and promoted activation of caspase -3/-7, induction of apoptosis [164].

**Fig. 3:**
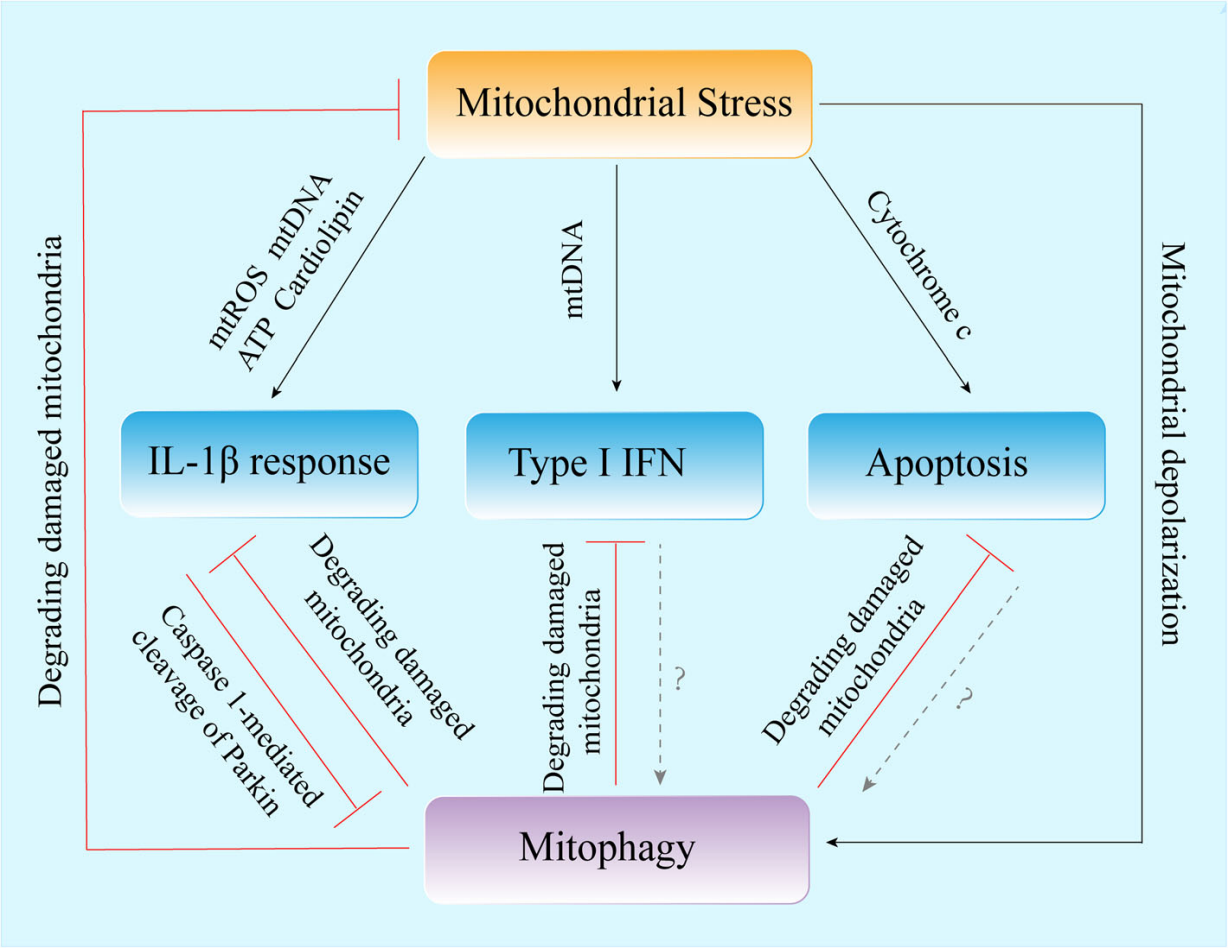
Functions of mitophagy in innate immune responses triggered by mitochondrial stress. Mitochondrial stress triggers the release of mtDNA, mtROS, ATP and cytochrome c into the cytosol and the externalization of cardiolipin, which lead to IL-1β secretion, type I IFN response and apoptosis. In addition, mitochondrial stress causes the dissipation of the mitochondrial membrane potential, which subsequently initiates the mitophagy process. Mitophagy attenuates the secretion of IL-1β, type I IFN responses and apoptosis by degrading dysfunctional mitochondria. In turn, activated caspase-1 can inhibit mitophagy by cleaving Parkin

Overall, mitophagy regulates immune responses triggered by mitochondrial stress, such as inflammasome activation, type I IFN responses and intrinsic apoptosis, through selective degrading dysfunctional mitochondria (Fig. 3). Furthermore, some pathogens can employ host mitophagy to avoid host immune defense. However, to date, our understanding of the role of mitophagy in the host innate immune and pathogenic immune evasion is limited, and further studies in vivo and in vitro are needed.

## Concluding remarks and future directions

The present work reviews the pivotal function of mitophagy in the regulation of innate immune responses induced by mitochondrial stress. Critically, mitophagy can attenuate the activation of inflammasome, type I IFN responses and apoptosis triggered by mitochondrial stress via clearing dysfunctional mitochondria. Notably, viruses or bacteria can employ host mitophagy to avoid host immune responses. Despite many efforts in the past to understand the role of pathogens infection in the regulation of mitophagy, we are still far from a clear understanding as to what the mechanisms of pathogens hijack host mitophagy. A more thorough understanding of the underlying molecular mechanisms by which pathogens trigger and regulate mitophagy, especially crosstalk between virulence factors of pathogens and host mitochondrial-specific proteins will provide new ways to develop therapeutic strategies to fight infection and related diseases.

## Data Availability

Not applicable.

## Abbreviations

PRRs: Pattern-recognition receptors
PAMPs: Pathogen-associated molecular patterns
TLRs: Toll-like receptors
NLRs: Nucleotide oligomerization domain (NOD)-like receptors
CLRs: C-type lectin receptors
RLRs: Retinoic acid-inducible gene I (RIG-I)-like receptors
NF-κB: Nuclear factor-κappa B
MAPKs: Mitogen-activated protein kinases
IFN: Interferon
IRFs: Interferon regulatory factors
mtDNA: Mitochondrial DNA
OXPHOS: Oxidative phosphorylation system
ROS: Reactive oxygen species
DAMPs: Damage-associated molecular patterns
mtROS: Mitochondrial ROS
NLRC4: Nod-like receptor CARD domain containing 4
NLRP3: Nod-like receptor pyrin domain containing 3
AIM2: Absence in melanoma 2
LPS: Lipopolysaccharide
TFAM: Mitochondrial transcription factor A
VDAC: Voltage-dependent anion channel
MPTP: Mitochondrial permeability transition pore
MAVS: Mitochondrial antiviral signaling proteins
ISGs: Interferon-stimulated genes
cGAS: Cyclic guanosine monophosphate–adenosine monophosphate synthase
cGAMP: Cyclic guanosine monophosphate–adenosine monophosphate
STING: Stimulator of interferon genes
TBK1: TANK-binding kinase 1
Mtb: *Mycobacterium tuberculosis*
BMDM: Bone marrow-derived macrophages
Bax: Bcl-2 associated-X protein
Bak: BCL-2 homologous antagonist/killer
TLR9: Toll-like receptor 9
CpG: Cytosine-guanosine
pDCs: Plasmacytoid dendritic cells
NASH: Nonalcoholic steatohepatitis
SIRS: Sterile systemic inflammatory response syndrome
MDVs: Mitochondria-derived vesicles
OMM: Outer mitochondrial membrane
PPAR: Peroxisome proliferator-activated receptor
HBHA: Heparin-binding hemagglutinin
KLK12: Kallikrein 12
ER: Endoplasmic reticulum
Pink1: Phosphatase and tensin homologue (PTEN)-induced putative kinase 1
Parkin: Parkin RBR E3 ubiquitin protein ligase
IMM: Inner mitochondrial membrane
PARL: Presenilin-associated rhomboid-like
OPA1: Optic atrophy 1
MFN1/2: Mitofusin 1/2
OPTN: Optineurin
NDP52: Nuclear domain 10 protein 52
DRP1: Dynamin-related protein 1
AMFR/ Gp78: Autocrine motility factor receptor
SMURF1: SMAD-specific E3 ubiquitin-protein ligase 1
MUL1: Mitochondrial E3 ubiquitin-protein ligase 1
SIAH1: Seven in absentia homolog 1
ARIH1: Ariadne RBR E3 Ub protein ligase 1
LIR: LC3-interacting region
BNIP3: BCL2 interacting protein 3
BNIP3L/NIX: BCL2 interacting protein 3 like
FUNDC1: FUN14 domain-containing protein 1
BCL2L13: BCL2 like 13
FKBP8: FKBP prolyl isomerase 8
RGC: Retinal ganglion cell
HIF1a: Hypoxia-inducible factor 1-alpha
PHB2: Prohibitin2
Atg16L1: Autophagy-related 16-like 1
HBV: Hepatitis B virus
HCV: Hepatitis C virus
MV-Edm: Measles virus of the Edmonston strain
HPIV3: Human parainfluenza virus type 3
TUFM: Tu translation elongation factor mitochondrial
HTNV: Hantavirus
*L. monocytogenes*: *Listeria monocytogenes*
RHDV: Rabbit haemorrhagic disease virus
